# Supplementary material for: Structural analysis of mtEXO mitochondrial RNA degradosome reveals tight coupling of nuclease and helicase components
Source: Nat Commun. 2018 Jan 8;9:97. doi: 10.1038/s41467-017-02570-5 (PMC5758563; doi:10.1038/s41467-017-02570-5)
Supplement: Supplementary file 1 — Supplementary Information [file 41467_2017_2570_MOESM1_ESM.pdf]

Supplementary Figures

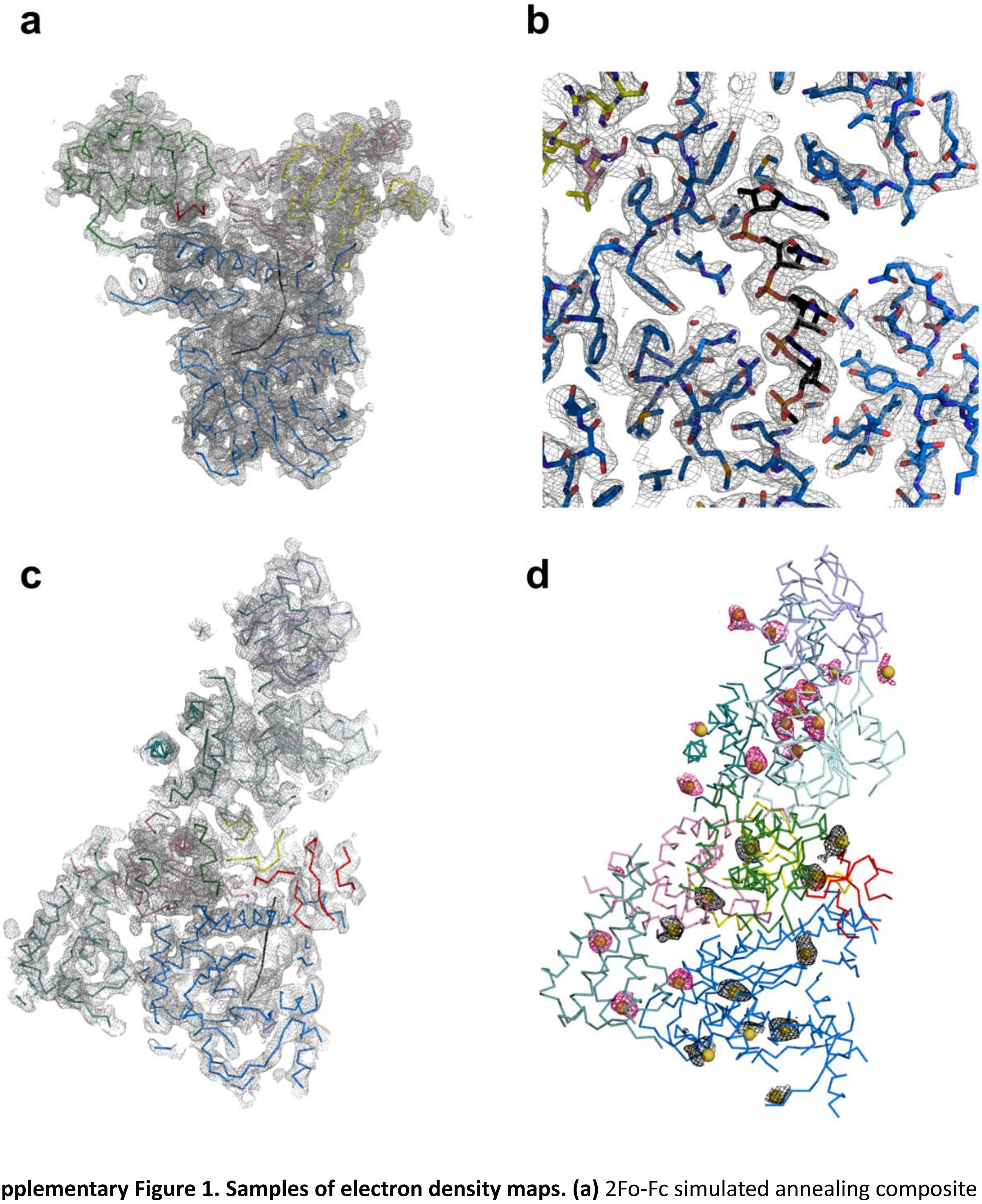

Supplementary Figure 1. Samples of electron density maps. (a) 2Fo-Fc simulated annealing composite omit map for *Cg*-Dss1$^{70\text{-}900}$ D477N crystal structure. The map is contoured at 1.0 σ and the protein is shown in wire representation. **(b)** Close-up view of the RNA-binding channel with structure shown as sticks. **(c)** 2Fo-Fc simulated annealing composite omit map of *Cg*-mtEXO (*Cg*-Dss1$^{70\text{-}900}$ D477N and *Cg*-Suv3$^{43\text{-}685}$) crystal structure contoured at 1.0 σ. **(d)** Positions of the selenomethionine residues within the *Cg*-mtEXO complex used to verify the tracing of its components and define the sequence register of the *Cg*-Suv3 N-terminal domain. Selenium atoms are shown as spheres. Anomalous difference maps that were calculated for the diffraction data for the crystals of mtEXO complexes with SeMet-Dss1$^{70\text{-}900}$ or SeMet-Suv3$^{43\text{-}685}$ are shown as black and magenta mesh, respectively. The maps are contoured at 3.0 σ.

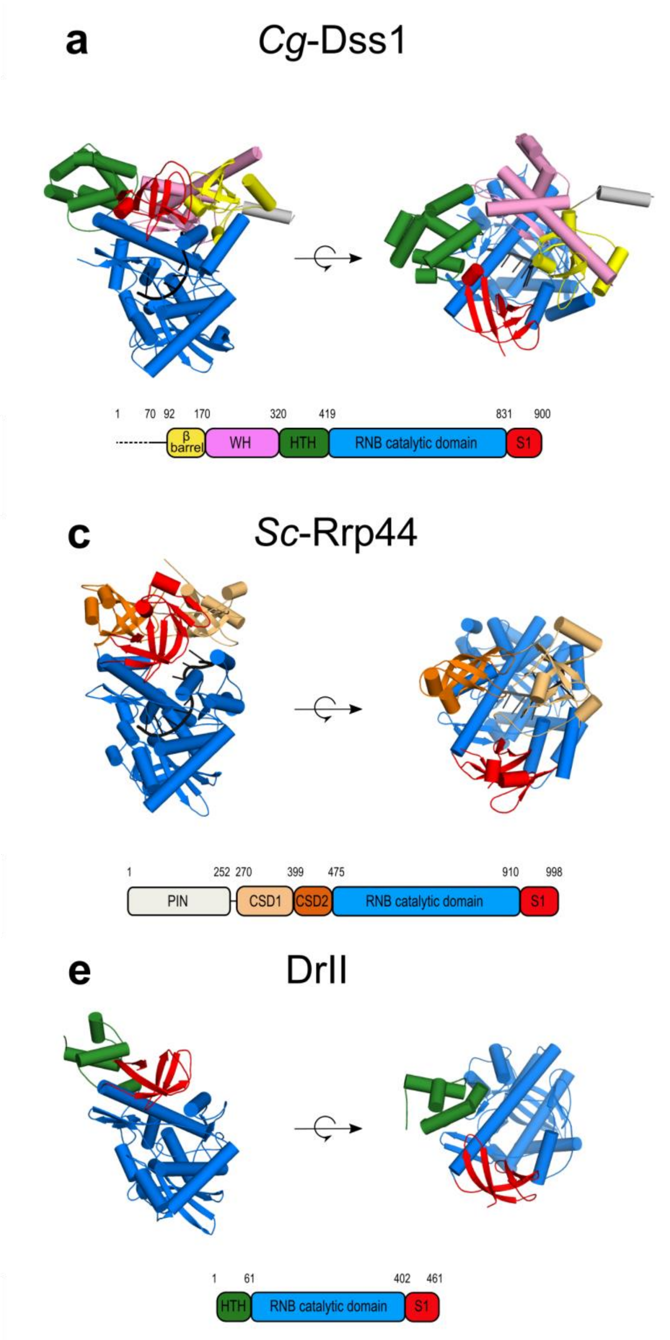

Supplementary Figure 2. Crystal structures of RNase II family members. (a) Dss1 from *C. glabrata*. Protein domains are colored according to the scheme below the structure and the N-terminal helix is shown in gray. RNA is shown in black. **(b)** RNase II from *E. coli* (PDB ID: 2IX1). The polyA$_{13}$ RNA chain is shown in black. **(c)** Rrp44 from *S. cerevisiae* (PDB ID: 2VNU), the nuclease component of the yeast RNA exosome. The polyA$_{10}$ chain is shown in black. **(d)** Dis3L2 from *M. musculus* (PDB ID: 4PMW). The polyU$_{14}$ RNA is represented in black. **(e)** DrII from *D. radiodurans* (PDB ID: 2R7F).

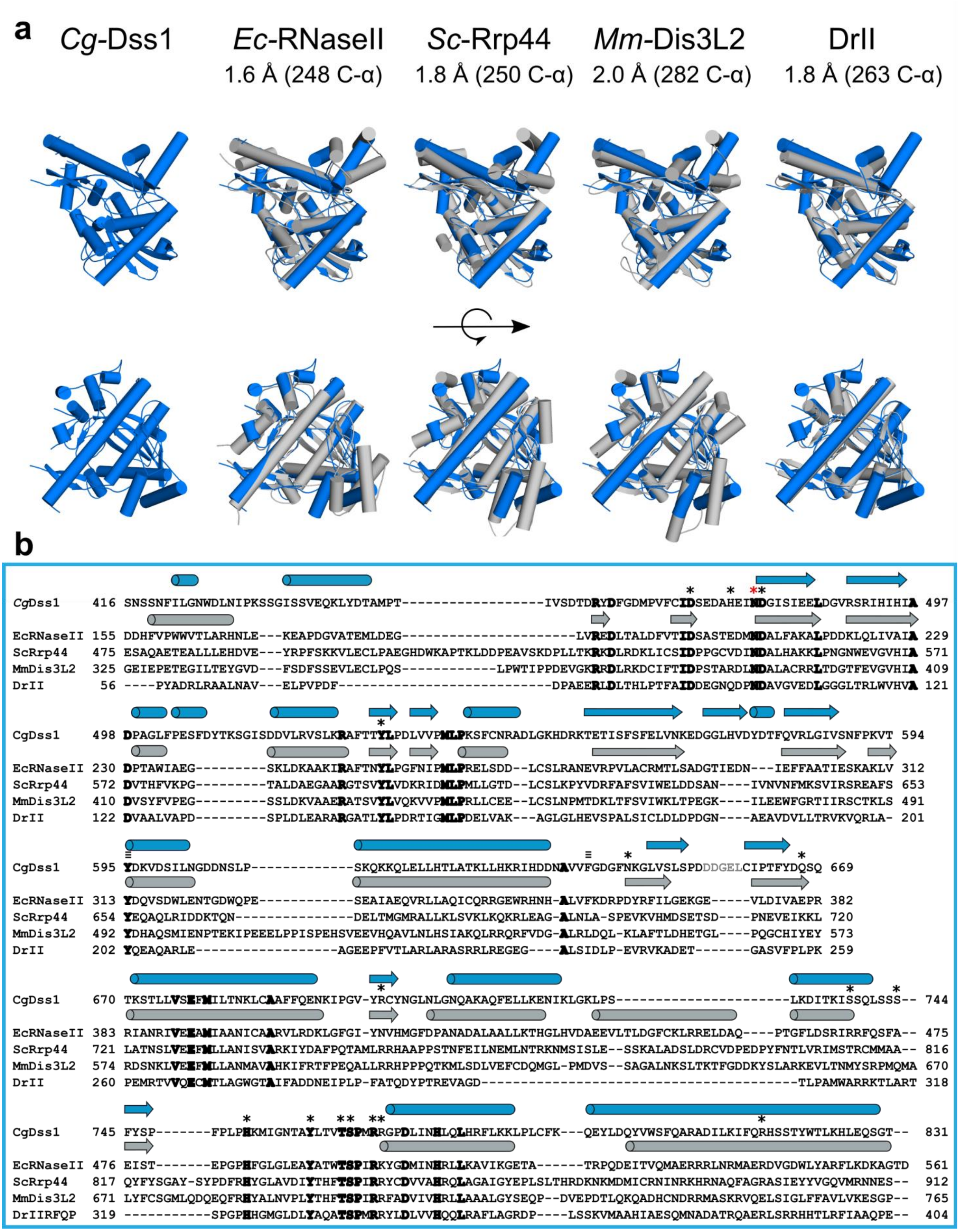

Supplementary Figure 3. Comparison of the RNB domains of *Cg*-Dss1 and other members of the RNase II family. (a) Superposition of the RNB domains of RNase II family members and *Cg*-Dss1. The root-mean-square deviation (r.m.s.d) values are given on top of each panel. **(b)** Structure-based sequence alignment RNB domains of RNase II proteins. For *Cg*-Dss1 and *Ec*-RNase II the secondary structures are indicated with arrows and barrels which correspond to β-sheets and α-helices, respectively. Residues that are involved in protein-RNA interactions are indicated as asterisks (*). Tyr530 and Phe640 residues that form the hydrophobic clamp are indicated as parallel lines. Asp477 which was substituted to Asn to inactivate the

enzyme is indicated with a red asterisk (*). Residues that are conserved among all RNase II-like enzymes are highlighted in bold.

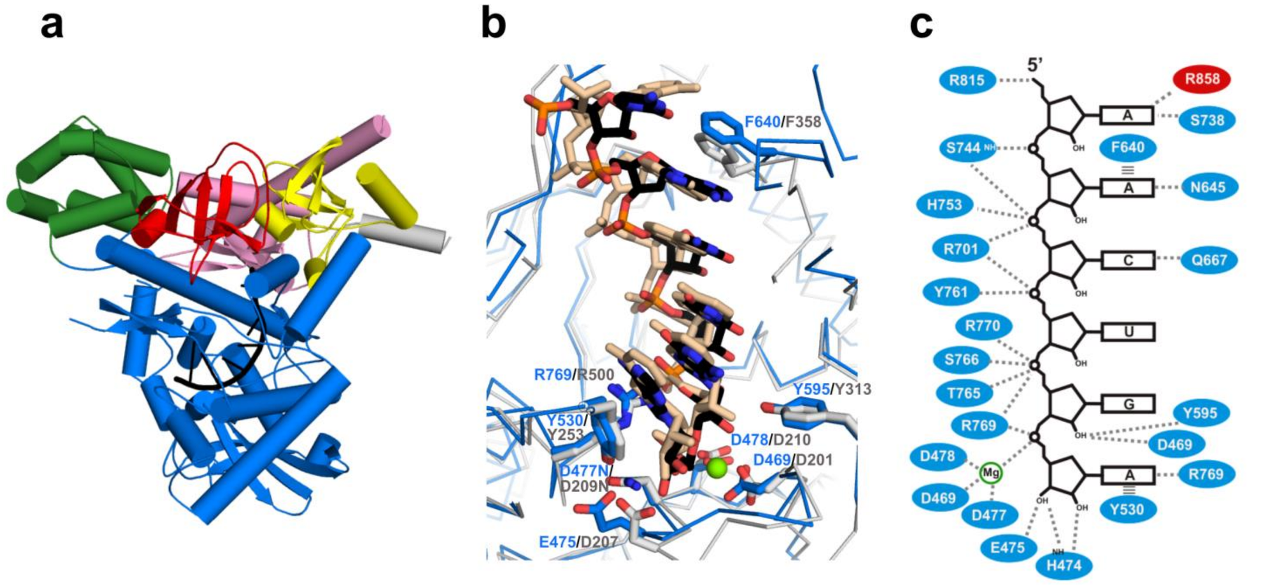

Supplementary Figure 4. RNA binding inside the RNB domain of Dss1 and comparison with RNase II. (a) *Cg*-Dss1$^{70\text{-}900}$ D477N structure (colored as in Fig. 1) with the RNA bound inside the RNB domain shown in black. **(b)** RNA binding by *Cg*-Dss1 and *Ec*-RNase II. The proteins are shown in wire representation (blue for *Cg*-Dss1 and gray for *Ec*-RNase II) and selected residues of the RNB domain involved in RNA cleavage and binding are represented as sticks. The RNA is shown as sticks – black for *Cg*-Dss1 and wheat for *Ec*-RNase II **(c)** Schematic representation of the *Cg*-Dss1–RNA interactions. Residues of the RNB domain are in blue, and the Arg858 residue of the S1 domain is in red. Hydrogen bonds are shown as dashed lines, and the stacking interactions with the aromatic clamp are shown as parallel lines. For detailed description see Supplementary Note 1.

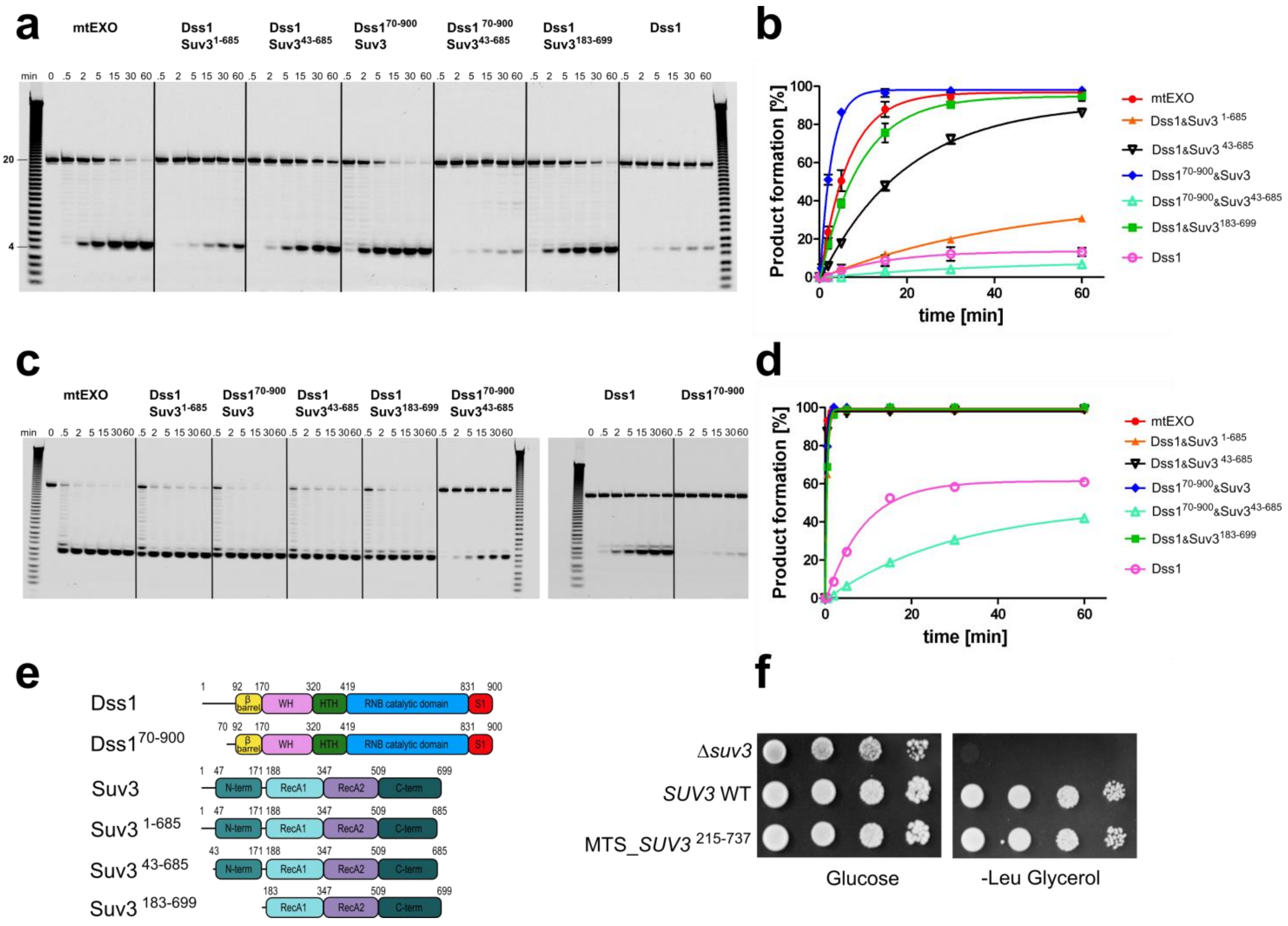

Supplementary Figure 5. Characterization of mtEXO deletion variants used for crystallization trials. **(a)** Exoribonuclease assay of *Cg*-mtEXO variants on T20-F ssRNA (pyrimidine-rich) substrate (see Supplementary Table 2 for sequence). The reaction products from the time-points that are indicated on top of the gel were analyzed by 20% TBE-urea PAGE scanned for fluorescent signal of the RNA. **(b)** Plot of RNA degradation kinetics (mean ± s.e.m. from three experiments) based on the results shown in (a). **(c)** Exoribonuclease assay of *Cg*-mtEXO variants on W20-F ssRNA (purine-rich) substrate (see Supplementary Table 2 for sequence). The reaction products from the time-points that are indicated on top of the gel were analyzed by 20% TBE-urea PAGE scanned for fluorescent signal of the RNA. **(d)** Quantification of the results in (c) from a single experiment. **(e)** Schematic representation of the truncated proteins used in the activity assay. **(f)** Respiratory competence of Δ*suv3 S. cerevisiae* strains that expressed WT and the allele of *Sc*-Suv3 with the deletion of the N-terminal domain (Suv3$^{215\text{-}737}$) but with the mitochondrial targeting sequence (MTS) retained.

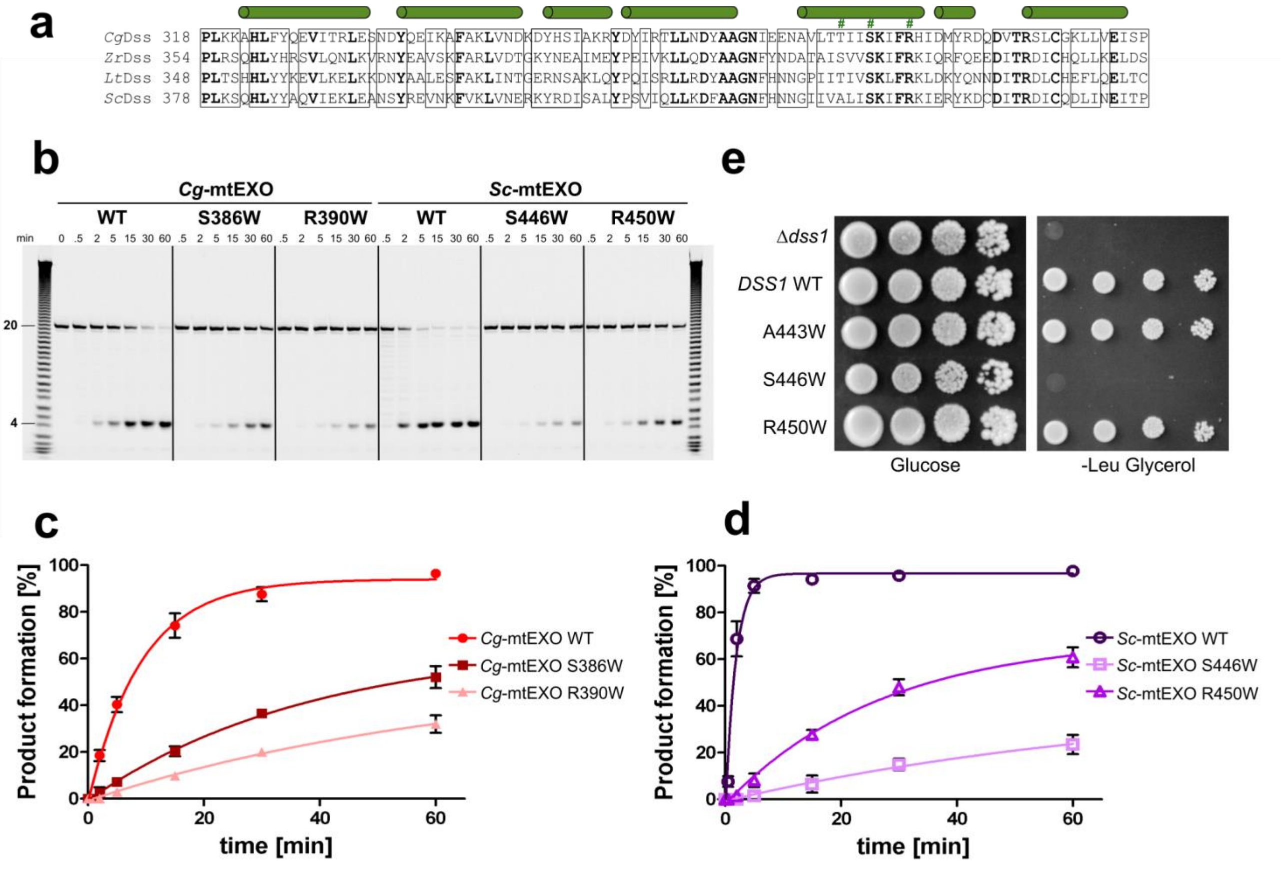

Supplementary Figure 6. Characterization of Dss1 variants with point substitutions in the HTH domain at the Dss1-Suv3 interface. (a) Sequence alignment of yeast Dss1 helix-turn-helix (HTH) domain performed with PROMALS3D [2]: *Cg* (*Candida glabrata*), *Zr* (*Zygosaccharomyces rouxii*), *Lt* (*Lachancea thermotolerans*), *Sc* (*Saccharomyces cerevisiae*). The barrels correspond to α-helices that are present in the *Cg*-Dss1$^{70-900}$ D477N structure. The residues of HTH domain of *Cg*-Dss1 that were substituted with tryptophan (Thr383, Ser386, Arg390) are indicated (*#*). **(b)** Exoribonuclease assay of *Cg* and *Sc* mtEXO complexes with tryptophan substitutions in the Dss1 HTH domain at the complex interface, on the T20-F ssRNA substrate (see Supplementary Table 2 for sequence). Samples from the time-course experiments were resolved on TBE-urea PAGE and the gels were scanned for fluorescent signals of the 6-FAM-labeled RNA. The protein variants that were used are indicated on top of the gel. **(c)** Quantification of the exoribonuclease activity of *Cg*-mtEXO wildtype (WT) and variants of the *Cg*-Dss1 HTH domain (mean ± s.e.m. from three experiments). **(d)** Quantification of the exoribonuclease activity of *Sc*-mtEXO WT and mutants of the *Sc*-Dss1 HTH domain (mean ± s.e.m. from three experiments). **(e)** Respiratory competence of Δ*dss1 S. cerevisiae* strains that expressed WT, A443W, S446W, and R450W substitution mutant alleles of *Sc*-Dss1.

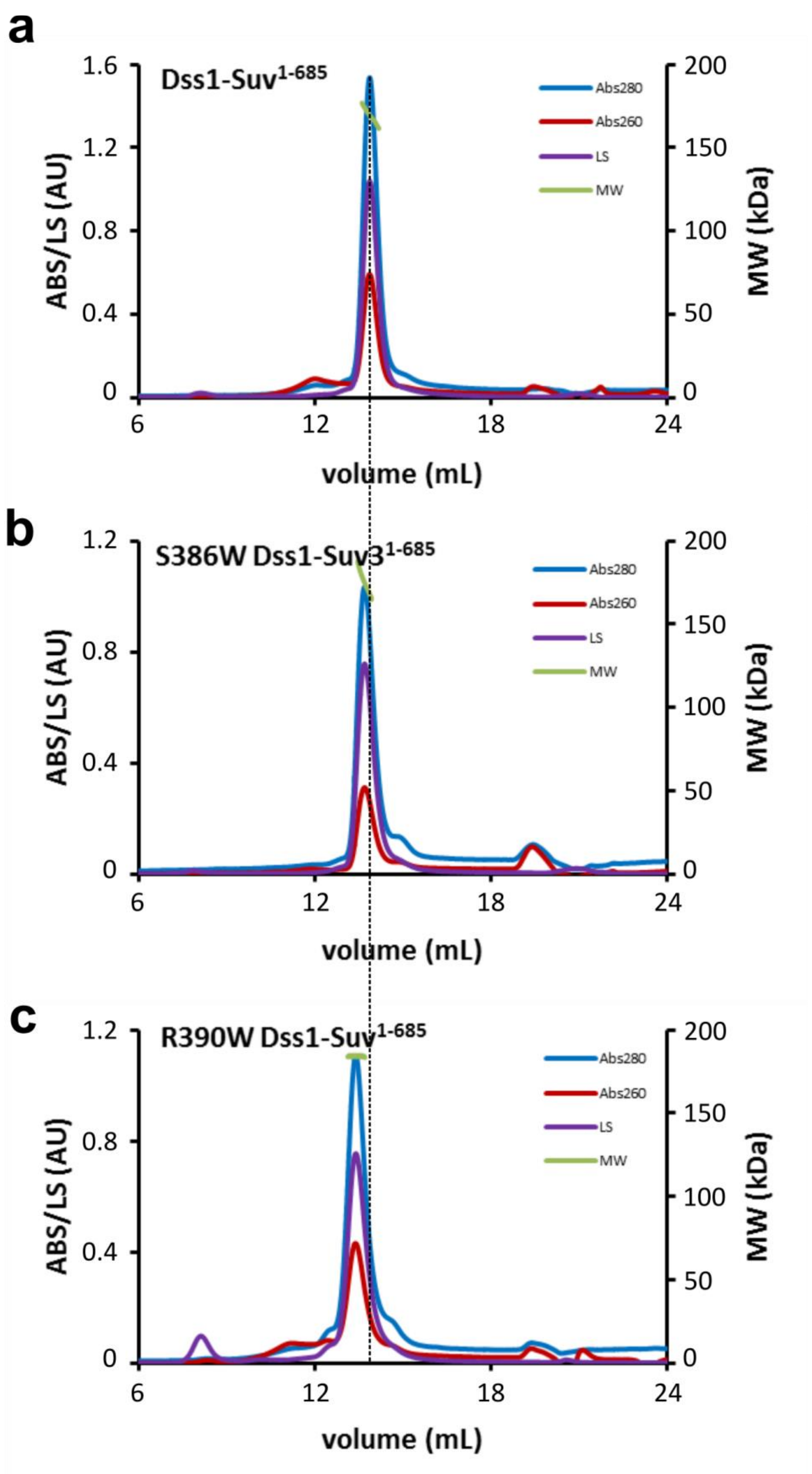

Supplementary Figure 7. Complex formation for mtEXO with point substitutions localized in the HTH domain of Dss1 (SEC-MALS). The absorbance signal at λ = 280 nm is indicated in blue. The absorbance signal at λ = 260 nm is indicated in red. The light scattering (LS) signal is indicated in violet. The calculated molecular weight (MW) is indicated in green. The position of the wildtype *Cg*-mtEXO peak is marked with a dashed line, indicating a shift of the elution volume for the tryptophan substitution variants. The theoretical molecular weights of the wild type complex is 181.1 kDa and calculated values based on light scattering were the following: (a) 169 kDa, (b) 184 kDa, (c) 175 kDa.

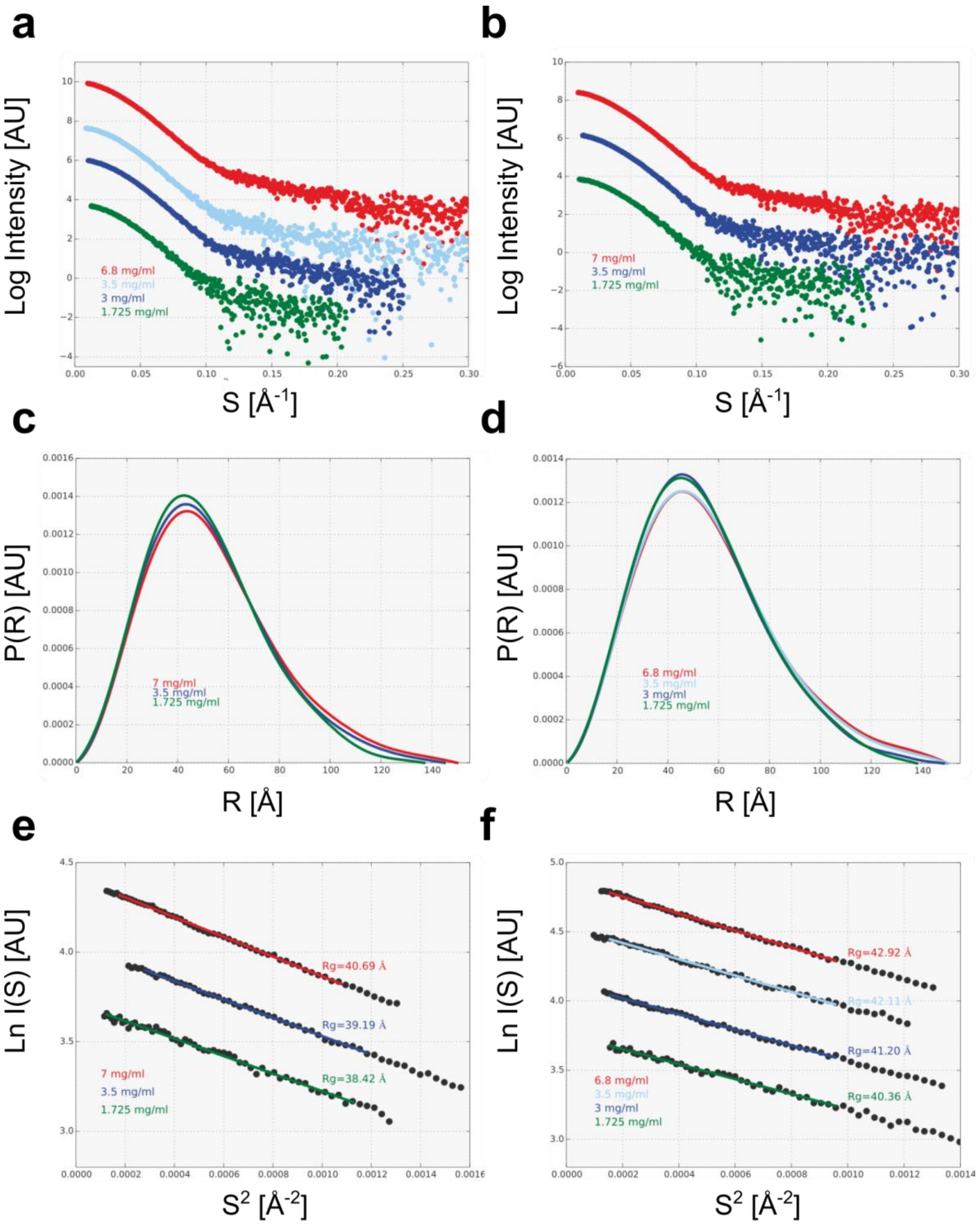

g

Sample	R_g (Å)	sR_g range	D_{max} (Å)	MW^{Vc}	V^{Porod} (Å^3)	MW^{Porod}
Cg-mtEXO 1.725 mg ml^-1	40.36 ± 0.51	0.49-1.28	138	149 kDa	299×10^3	187 kDa
Cg-mtEXO 3 mg ml^-1	41.20 ± 0.36	0.41-1.29	148	171 kDa	295×10^3	184 kDa
Cg-mtEXO 3.5 mg ml^-1	42.11 ± 0.31	0.36-1.29	150	166 kDa	301×10^3	188 kDa
Cg-mtEXO 6.8 mg ml^-1	42.92 ± 0.26	0.46-1.29	150	169 kDa	304×10^3	190 kDa
Cg-Dss1-Suv3$^{183-699}$ 1.725 mg ml^-1	38.42 ± 0.56	0.41-1.29	129	146 kDa	278×10^3	174 kDa
Cg-Dss1-Suv3$^{183-699}$ 3.5 mg ml^-1	39.19 ± 0.22	0.59-1.22	145	151 kDa	263×10^3	164 kDa
Cg-Dss1-Suv3$^{183-699}$ 7 mg ml^-1	40.69 ± 0.26	0.47-1.29	150	159 kDa	280×10^3	175 kDa

Supplementary Figure 8. Structural parameters from SAXS data. (a, c, e) SAXS scattering curves, pair distance distribution function P(R) and Guinier plot obtained for the full-length *Cg*-mtEXO complex. **(b, d, f)** SAXS scattering curves, pair distance distribution function P(R) and Guinier plot obtained for *Cg*-Dss1-Suv3$^{183-699}$. **(g)** Structural parameters obtained from the experimental SAXS data for the full-length *Cg*-mtEXO and *Cg*-Dss1-Suv3$^{183-699}$. R_g, radius of gyration. D_{max}, maximum diameter. MW^{Vc}, molecular weight calculated using volume of correlation method implemented in Scatter program [3]. MW^{Porod}, molecular

weight calculated from the formula: MW= V^{porod} x 1600^-1. mtEXO theoretical molecular weight: 183 kDa. *Cg*-Dss1-Suv3$^{183\text{-}699}$ theoretical molecular weight: 161.8 kDa.

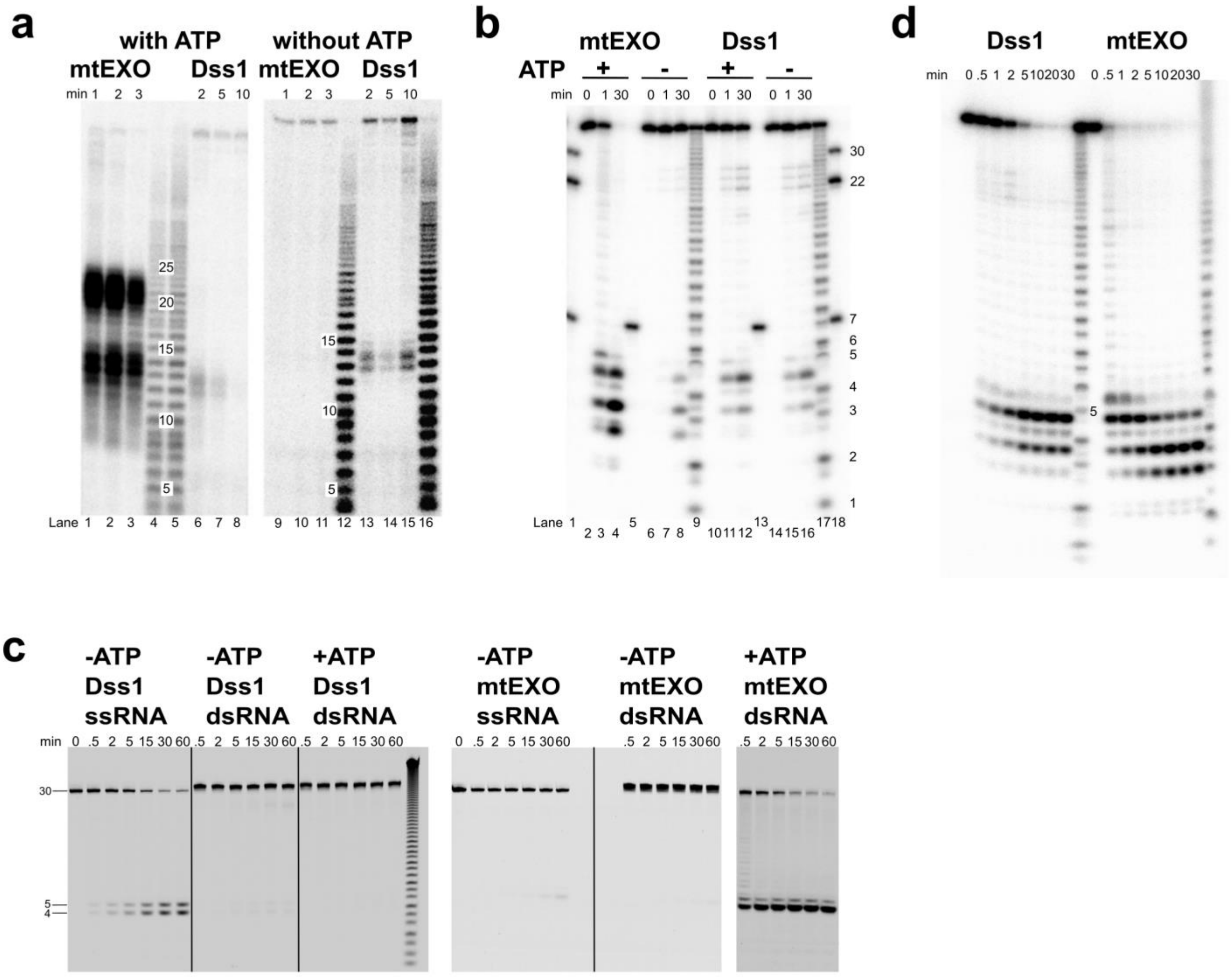

Supplementary Figure 9. The effect of adenosine triphosphate hydrolysis on the activity of *Cg*-Dss1 and *Cg*-mtEXO. (a) RNAse I RNA footprinting experiment with a 79-nt ^{32}P body-labeled L1 RNA (see Supplementary Fig. 10 for sequence) and full-length *Cg*-mtEXO or *Cg*-Dss1. The experiments were performed in the presence (lanes 1-3 and 6-8) or absence (lanes 9-11 and 13-15) of ATP. This was followed by addition of EDTA to stop the *Cg*-mtEXO and *Cg*-Dss1 activities, and addition of RNAse I to digest RNA that was not bound by the proteins. The RNA ladder was obtained after the alkaline hydrolysis of 5′ ^{32}P-labeled L1 RNA for 27 min (lanes 4 and 12) or 20 min (lanes 5 and 16). **(b)** Multiple turnover RNA degradation of R36 ssRNA by wildtype fl-*Cg*-mtEXO or fl-*Cg*-Dss1 in the presence or absence of ATP for the indicated times. Reference lanes contain 5′ ^{32}P-labeled RNAs of known lengths: 30-mer and 22-mer RNAs (lanes 1 and 18), 7-mer RNA (lanes 1, 5, 13, and 18). The alkaline hydrolysis ladder of the 5′ ^{32}P-labeled R36 RNA is in lanes 9 and 17. The major bands, corresponding to 5′ and 2′-3′ cyclic phosphate fragments, are numbered. The less intense bands represent faster migrating 5′ phosphates with opened 2′ or 3′ phosphates. **(c)** Nuclease activity of fl-*Cg*-mtEXO on dsRNA. (oligonucleotide W30-F was used as a ssRNA substrate, dsRNA with a 3′ overhang was prepared by annealing oligonucleotides W30-F and T20; see Supplementary Table 2 for sequence). Time-course reactions were analyzed by 20% TBE-urea PAGE and scanned for fluorescent signal of the RNA. **(d)** Single turnover exoribonuclease activity of fl-*Cg*-mtEXO complex for 5′ ^{32}P-labelled R36 RNA (see Supplementary Fig. 10b for sequence). Time-course reactions were analyzed by 18% TBE-urea PAGE (plot of R36 RNA degradation kinetics with mean ± s.e.m. from three experiments is shown in Fig. 5b).

a

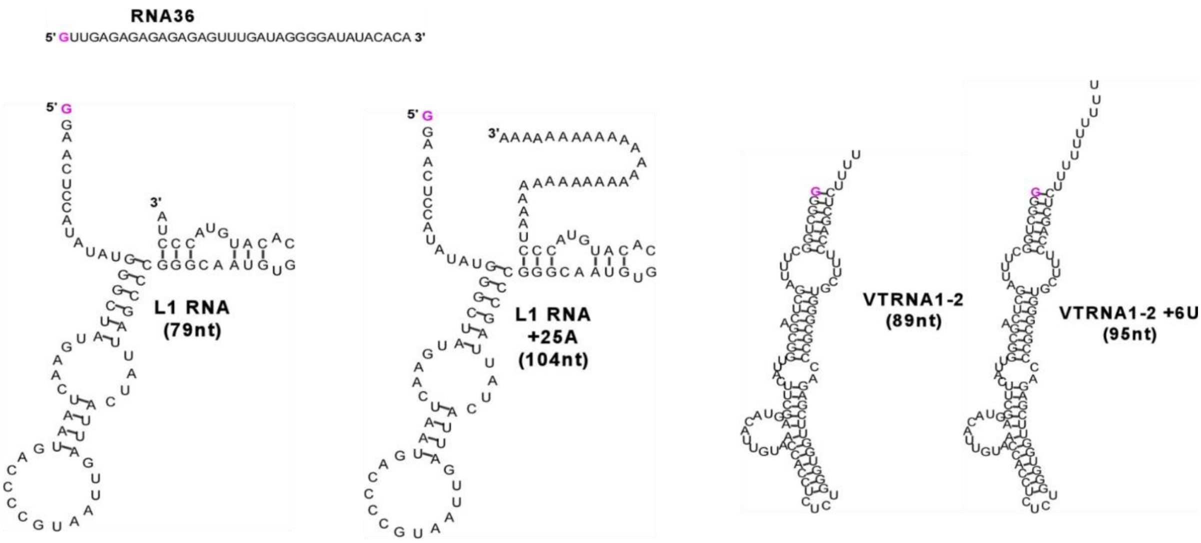

b

Oligo name	Sequence	Template DNA (forward strand)
L1	GGAACUCCAUAUAUGGGCUAUGAACUAAUGACCCCGUAAUUGAUUACUAUUAGCCCGGGCAAUGUGCACAUGUACCCUA	GTGAG**AGATGTAATACGACTCACTATAGG**GGAACTCCATATATGGGCTATGAACTAATGACCCCGTAATTGATTACTATTAGCCCGGGCAATGTGCACATGTACCCTA
L1 25A	GGAACUCCAUAUAUGGGCUAUGAACUAAUGACCCCGUAAUUGAUUACUAUUAGCCCGGGCAAUGUGCACAUGUACCCUAAAAAAAAAAAAAAAAAAAAAAAAA	GTGAG**AGATGTAATACGACTCACTATAGG**GGAACTCCATATATGGGCTATGAACTAATGACCCCGTAATTGATTACTATTAGCCCGGGCAATGTGCACATGTACCCTAAAAAAAAAAAAAAAAAAAAAAAAAA
VTRNA1-2	GGCUGGCUUUAGCUCAGCGGUUACUUCGAGUACAUUGUAACCACCUCUCUGGGUGGUUCGAGACCCGCGGGUGCUUUCCAGCUCUUUU	**AGATGTAATACGACTCACTATAGG**GCTGGCTTTAGCTCAGCGGTTACTTCGAGTACATTGTAACCACCTCTCTGGGTGGTTCGAGACCCGCGGGTGCTTTCCAGCTCTTTT
VTRNA1-2 6U	GGCUGGCUUUAGCUCAGCGGUUACUUCGAGUACAUUGUAACCACCUCUCUGGGUGGUUCGAGACCCGCGGGUGCUUUCCAGCUCUUUUUUUUUU	**AGATGTAATACGACTCACTATAGG**GCTGGCTTTAGCTCAGCGGTTACTTCGAGTACATTGTAACCACCTCTCTGGGTGGTTCGAGACCCGCGGGTGCTTTCCAGCTCTTTTTTTTTT
R36	GUUGAGAGAGAGAGAGUUUGAUAGGGGAUAUACACA	

Supplementary Figure 10. 5′ ^{32}P-labelled RNA oligonucleotides used for RNAse I protection assay and RNA degradation assays. (a) The structures of RNA substrates that form internal base-pairs. The graphs were prepared using the RNA fold server [4]. **(b)** Sequences of the RNA substrates and DNA templates used for transcription. The 5′ ^{32}P-label is highlighted in pink. The T7 consensus promoter sequence with stabilizing nucleotides in PCR-amplified DNA templates for *in vitro* run-off transcription are in bold.

Supplementary Table 1. *Cg*-Dss1 and *Cg*-Suv3 expression constructs used in this study. All constructs express a fusion of the protein of interest with six-histidine tag (His_6) and maltose-binding protein (MBP).

Expression constructs	Description
pDEST-His_6MBP-fl-*Cg*-Dss1	Full-length *Candida glabrata (Cg)* Dss1
pDEST-His_6MBP-*Cg*-Dss1$^{70\text{-}900}$	N-terminal truncation of *Cg*-Dss1
pDEST-His_6MBP-*Cg*-Dss1$^{70\text{-}900}$ D477N	N-terminal truncation of *Cg*-Dss1 with point mutation at the active site
pDEST-His_6MBP-fl-*Cg*-Suv3	Full-length *Cg*-Suv3
pDEST-His_6MBP-fl-*Cg*-Dss1 R390W	Full-length *Cg*-Dss1 with point mutation of the Dss1-HTH domain
pDEST-His_6MBP-fl-*Cg*-Dss1 S386W	Full-length *Cg*-Dss1 with point mutation of the Dss1-HTH domain
pDEST-His_6MBP-*Cg*-Suv3$^{1\text{-}685}$	*Cg*-Suv3 with the 14 amino acid (aa) C-terminal truncation
pDEST-His_6MBP-*Cg*-Suv3$^{43\text{-}685}$	*Cg*-Suv3 with the 42 aa N-terminal and 14 aa C-terminal truncations
pDEST-His_6MBP--*Cg*-Suv3$^{183\text{-}699}$	*Cg*-Suv3 with the 182 aa truncation of the N-terminal domain
pDEST-His_6MBP-fl-*Sc*-Dss1	Full-length *Saccharomyces cerevisiae (Sc)* Dss1
pDEST-His_6MBP-fl-*Sc*-Suv3	Full-length *Sc*-Suv3
pDEST-His_6MBP-fl-*Sc*-Dss1 R450W	Full-length *Sc*-Dss1 with point mutation in the Dss1-HTH domain
pDEST-His_6MBP-fl-*Sc*-Dss1 S446W	Full-length *Sc*-Dss1 complex with point mutation in the Dss1-HTH domain

Supplementary Table 2. RNA oligonucleotides used for activity assays and crystallization of the mtEXO complex (20Tx and 12Bx are the top and bottom strands, respectively). "F" denotes 6-carboxyfluorescein (6-FAM) modification of the 5' end of the RNA molecule.

Oligo name	Sequence (5' -3')
T20-F	**F**-CAAACUCUCUCUCUCUCAAC
T20	CAAACUCUCUCUCUCUCAAC
W20-F	**F**-AGAGAGUUUGAGAGAGAGAG
W30-F	**F**-GUUGAGAGAGAGAGAGUUUGAGAGAGAGAG
W50-F	**F**-GUUGAGAGAGAGAGAGUUUGAGAGAGAGAGAGAGAGUUUGAGAGAGAGAG
20Tx	AUAAAAUAAUAUUCUUAAUU
12Bx	AUAUUAUUUUAU

Supplementary Table 3. Oligonucleotides used for plasmid preparation, yeast strain construction and site-directed mutagenesis (the mutated codon is underlined).

Oligo name	Sequence (5′-3′)
DSS1_slicL	GCCTGCAGGTCGACTCTAGAGGATCTTGTGTATCGGAATCCGGCTC
DSS1_slicR	AATTCGAGCTCGGTACCCGGGGATCTAACACCATCGCAGCAACGAG
SUV3_slicL	GCCTGCAGGTCGACTCTAGAGGATCTTTCCAACGCAAGCAGTGTACG
SUV3_slicR	AATTCGAGCTCGGTACCCGGGGATCGGTTCTGGCAGTCGATACAATG
DSS1_A	GTTTACAAATTGAATCGGATGACTC
DSS1_D	TTTATAGTGGAGAAGAGAACCATCG
SUV3_A	TCAGAACACAATGTCCTTATTGAAA
SUV3_D	TATATTTTACTGCCCTTTGCTCAAC
A443W_forward	CACAATAACGGAATTATAGTATGGTTGATCTCAAAAATATTCAGAAAGATAGAACGC
A443W_reverse	GCGTTCTATCTTTCTGAATATTTTTGAGATCAACCATACTATAATTCCGTTATTGTG
S446W_forward	CACAATAACGGAATTATAGTAGCTTTGATCTGGAAAATATTCAGAAAGATAGAACGC
S446W_reverse	GCGTTCTATCTTTCTGAATATTTTCCAGATCAAAGCTACTATAATTCCGTTATTGTG
R450W_forward	CGGAATTATAGTAGCTTTGATCTCAAAAATATTCTGGAAGATAGAACGCTATAAGG
R450W_reverse	CCTTATAGCGTTCTATCTTCCAGAATATTTTTGAGATCAAAGCTACTATAATTCCG
MdSUV3_forward	GAAAGCATATTACCACAGCGAGGTTGACATAACAAATCCAGCAG
MdSUV3_reverse	CTGCTGGATTTGTTATGTCAACCTCGCTGTGGTAATATGCTTTC
Cg-DSS1D477N_forward	GACTCCGAAGATGCTCACGAAATAAATGATGGCATTTCAATTGAGGAG
Cg-DSS1D477N_reverse	CTCCTCAATTGAAATGCCATCATTTATTTCGTGAGCATCTTCGGAGTC
Cg-DSS1R390W_forward	ATAAGTAAAATATTCTGGCATATCGACATGTAT
Cg-DSS1R390W_reverse	ATACATGTCGATATGCCAGAATATTTTACTTAT
Cg-DSS1S386W_forward	GTCTTAACAACAATAATATGGAAAATATTCAGGCATATC
Cg-DSS1S386W_reverse	GATATGCCTGAATATTTTCCATATTATTGTTGTTAAGAC
Sc-DSS1R450W_forward	GATCTCAAAAATATTCTGGAAGATAGAACGCTATA
Sc-DSS1R450W_reverse	TATAGCGTTCTATCTTCCAGAATATTTTTGAGATC
Sc-DSS1S446W_forward	GAATTATAGTAGCTTTGATCTGGAAAATATTCAGAAAGATAGA
Sc-DSS1S446W_reverse	TCTATCTTTCTGAATATTTTCCAGATCAAAGCTACTATAATTC
Cg-DSS1$^{70-900}$_forward	GAATCTTTATTTTCAGGGCGCCATGTTTATTATCAACTCGGACTTTCATC
Cg-DSS1$^{70-900}$_reverse	GATGAAAGTCCGAGTTGATAATAAACATGGCGCCCTGAAAATAAAGATTC
Cg-SUV3$^{183-699}$_forward	CTTTATTTTCAGGGCGCCATGGTGGATTTTTCTAACCCAGCA
Cg-SUV3$^{183-699}$_reverse	TGCTGGGTTAGAAAAATCCACCATGGCGCCCTGAAAATAAAG
Cg-SUV3$^{1-685}$_forward	CACCTGAAGAGAAACCCCTACTAATAAATCCGCAAGAAATTCATATCG
Cg-SUV3$^{1-685}$_reverse	CGATATGAATTTCTTGCGGATTTATTAGTAGGGGTTTCTCTTCAGGTG
Cg-SUV3$^{43-685}$_forward	CTTTATTTTCAGGGCGCCATGATATACACCACAGATAAAGAG
Cg-SUV3$^{43-685}$_reverse	CTCTTTATCTGTGGTGTATATCATGGCGCCCTGAAAATAAAG

Supplementary Note 1

RNA binding inside the catalytic domain of Dss1

In the refined structure of *Cg*-Dss1, we observed electron density for a 6-nt RNA fragment inside the RNB channel (Supplementary Figure 4a, b). RNA was not included in the crystallization and likely co-purified with the protein that was produced in *E. coli*. Therefore, an arbitrary RNA sequence was used for model building. *Cg*-Dss1 that contained an active site mutation D477N was used for crystallization, and the RNA that was bound in the catalytic channel likely could not be digested by the enzyme and thus was protected from degradation by other nucleases during cell

lysis and purification. This was similar to RNase II, which was also crystallized as a complex with a 13 nt ssRNA that remained bound by the protein throughout purification [5].

In the *Cg*-Dss1 structure, the electron density for RNA is only visible in the channel of the RNB domain, and no interactions are observed between the nucleic acid and smaller domains that decorate the catalytic core, aside from the Arg858 residue of the S1 domain (Supplementary Fig. 4c). The 2′-OH groups of nucleotides 1 and 2 (numbered from the 3′-end of the substrate) form hydrogen bonds with side chains of conserved residues of the RNB domain (His474, Asp469, and Tyr595 in *Cg*-Dss1), leading to specific RNA binding (Supplementary Fig. 4b, c). The 3′ end of the RNA in the Dss1 structure reaches the active site of the enzyme, which is composed of residues that are highly conserved in Dss1 and the RNR family (Supplementary Fig. 3b). We observed electron density for a single catalytic Mg^{2+} ion that is coordinated by Asp469 and Asp478. Similar ion coordination was observed for RNase II [5]. The RNA is stabilized by an aromatic clamp that consists of sidechains of Tyr530 and Phe640 that form stacking interactions with the first and fifth bases from the 3′ end.

Supplementary Note 2

Exoribonuclease activity of mtEXO deletion variants

In the crystallization trials we tested various deletion variants of *Cg*-Dss1 and *Cg*-Suv3 which were obtained by removal of short protein fragments that were predicted to be disordered. The activity of these variants was first tested on T20-F ssRNA (pyrimidine-rich) substrate (Supplementary Fig. 5a, b; see Supplementary Table 2 for sequence). We mixed the purified mutant complexes with RNA substrates that were labeled on their 5′ end with the fluorescent dye 6-carboxyfluorescein (6-FAM; Supplementary Table 2). The reactions were initiated by rapidly mixing proteins with RNAs in the presence of Mg^{2+} and ATP. Aliquots were withdrawn at the indicated time points, and the products of the degradation reaction were visualized by

polyacrylamide gel electrophoresis (PAGE). We first tested the activity of the full-length *Cg*-Dss1 which had low activity (reaction half-life = 9.9 min). The N-terminal truncation of *Cg*-Dss1 in complex with *Cg*-Suv3 (complex *Cg*-Dss1$^{70\text{-}900}$-Suv3) resulted in greater activity (reaction half-life = 1.8 min) compared with fl-*Cg*-mtEXO (half-life = 4.5 min). The N-terminus of Dss1 may participate in substrate recruitment (*Cg*-Dss1 with N-terminal deletion is inactive, see below). Within the mtEXO complex, substrate recruitment and feeding into the catalytic channel is performed by Suv3, so the N-terminus of Dss1 is no longer required and may in fact compete for RNA binding with Suv3. This would explain why deletion of the N-terminus of *Cg*-Dss1 leads to greater activity of the *Cg*-mtEXO complex. The C-terminal truncation of 14 residues of *Cg*-Suv3 (complex *Cg*-Dss1-Suv3$^{1\text{-}685}$) led to a large reduction of activity (half-life = 31.4 min). However, this deletion, combined with an N-terminal *Cg*-Suv3 truncation (complex *Cg*-Dss1-Suv3$^{43\text{-}685}$), resulted in the rescue of activity (half-life = 13.7 min). These results indicate that the termini of *Cg*-Suv3, which are predicted to be disordered, exert a complex influence on activity of the complex, both stimulatory (C-terminus) and inhibitory (N-terminus). The activity of the crystallized complex (*Cg*-Dss1$^{70\text{-}900}$-Suv3$^{43\text{-}685}$) was low, with a reaction half-life of 28.2 min. The *Cg*-mtEXO variant with a deletion of the N-terminal domain of Suv3 (*Cg*-Dss1- Suv3$^{183\text{-}699}$) retained full exoribonuclease activity (half-life = 6.4 min).

Next, we have tested the exoribonuclease activities of these variants using a purine-rich substrate W20-F (Supplementary Fig. 5c, d; see Supplementary Table 2 for sequence). Interestingly, the purine-rich substrate was degraded much more efficiently by mtEXO (half-life = 0.13 min) than the pyrimidine-rich RNA (half-life = 4.5 min). This indicates some substrate sequence preference of mtEXO that may have consequences for the physiological role of this complex. The activity of the crystallized variant was also higher than in the case of T20-F substrate (half-life = 21 min). Full-length *Cg*-Dss1 digested 61% of the RNA (half-life = 6.1 min). The crystallized *Cg*-Dss1$^{70\text{-}900}$ variant was inactive. This suggests that the N-terminus of Dss1 plays a role in recruiting the substrate. The

importance of this region is further supported by the fact that Dss1 proteins from various fungal species show strong sequence conservation of the region that corresponds to residues 1-43 in *Cg*-Dss1.

Supplementary Note 3

Characterization of mtEXO variants with substitutions in Dss1 HTH domain

Our *Cg*-mtEXO crystal structure revealed protein-protein interfaces between Suv3 and Dss1. To verify the importance of these contacts, we introduced bulky tryptophan residues to disrupt the protein-protein interface. We substituted Ser386 and Arg390 in fl-*Cg*-Dss1. Both residues are located in the HTH domain that forms contacts with the non-canonical B-α-1′ helix of the RecA1 domain of *Cg*-Suv3 (Fig. 2c). The size-exclusion chromatography–multiangle light scattering (SEC-MALS) elution profiles of *Cg*-Dss1 S386W-Suv$^{1\text{-}685}$ and *Cg*-Dss1 R390W-Suv3$^{1\text{-}685}$ (fl-*Cg*-Suv3 was unstable in the SEC experiments) complexes contained single peaks with measured molecular weights of 184 and 175 kDa, respectively, indicating that neither substitution influenced formation of the *Cg*-mtEXO complex (Supplementary Fig. 7). However, the elution volume was different for mutant complexes, implying that their overall structure was altered.

To test the effect of the substitutions on *Cg*-mtEXO activity, we performed exoribonuclease activity assays. Wildtype *Cg*-mtEXO catalyzed efficient hydrolysis of the 20T-F RNA substrate. The RNA degradation half-life was 6.4 min, and the reaction continued to near completion (Supplementary Fig. 6b, c). The *Cg*-mtEXO variants with tryptophan substitutions exhibited significantly lower RNA degradation kinetics of the T20-F RNA substrate, with substrate half-life equal to 26.6 and 46.2 min for the S386W and R390W variants, respectively. Although the *Cg*-Dss1 R390W and S386W variants could still form a complex with *Cg*-Suv3, perhaps involving the interface between the C-terminal domain of *Cg*-Suv3 and a long kinked α-helix of the WH domain,

the results indicate that the two proteins within the complex were not properly oriented for efficient RNA hydrolysis.

The orthologous Dss1 protein sequences of *C. glabrata* and *S. cerevisiae* show significant conservation (45% identity, 62% similarity). The well-established model species *S. cerevisiae* can thus be used to study the phenotypic effects of Dss1 substitutions at sites that form the interface with Suv3. We studied variants of *S. cerevisiae* Dss1 with tryptophan substitutions of Ser446 and Arg450 (equivalents of Ser386 and Arg390 in *C. glabrata* protein; see Supplementary Fig. 6a for sequence alignment of yeast Dss1 HTH domain). The A443W variant was used as a control. Ala443 corresponds to *C. glabrata* Thr383, which is at the edge of the interface. This residue is not conserved among fungal species. We assumed that its substitution would not affect activity of the complex. Variants of the *S. cerevisiae* protein were first tested *in vitro* (Supplementary Fig. 6b, d). We observed a general decrease in exoribonucleolytic activity of the mutants, which corroborated the results that were obtained with the *C. glabrata* enzyme. Wildtype *Sc*-mtEXO showed RNA degradation, with a half-life of 1.2 min, whereas the S446W and R450W mutants had RNA degradation half-life of 52.6 and 18.9 min, respectively. We next tested the respiratory competence of *S. cerevisiae* strains that carried A443W, S446W, and R450W by assaying growth on glycerol, which is a non-fermentable carbon source (Supplementary Fig. 6e). As expected and based on previous studies [6], the Dss1 knockout is strictly respiratory deficient, and the phenotype can be complemented by the wildtype gene (Supplementary Fig. 6e). The strain transformed with S446W variant of *Sc*-Dss1 was also unable to grow on glycerol at either normal or elevated temperature, corresponding to a complete loss-of-function phenotype. However, neither the A443W nor R450W substitution had an observable effect on respiratory growth (Supplementary Fig. 6e). These results agree with the results of the activity assays, in which S446W had a more severe defect than R450W. The results also imply that the lower activity of R450W was still sufficient for complementation (Supplementary Fig. 6e). In summary, our *in vitro* and *in vivo*

studies confirmed the importance of the Dss1 HTH-Suv3 RecA1 interface, which is in agreement with our *Cg*-mtEXO structure.

Supplementary Note 4

Extended analysis of the RNAse I footprinting results

In our RNAse I RNA footprinting assays we observed ATP-dependent protection of 13-15 and 20-24 nt RNA fragments by fl-*Cg*-mtEXO (Supplementary Fig. 9a). These fragments are both longer and shorter than expected based on the complex crystal structure and the RNA channel length estimation presented in Fig. 4. We propose that the protection of the longer fragment (20-24 nt) resulted from additional interactions of the RNA around the entry site into the fl-*Cg*-Suv3 structure. In the case of the 13-15 nt protected RNA regions that were slightly shorter than the length of the bound RNA predicted based on the structural data, we assume that this difference is attributable to “breathing” of the complex, particularly conformational changes of the Suv3 subunit. Additionally, the RNase I digestion in the footprinting experiments was performed under conditions in which ATP hydrolysis and RNA translocation did not occur (after addition of EDTA to inhibit the activities of *Cg*-mtEXO and *Cg*-Dss1). Therefore, back-and-forth translocations of RNA were possible, thus shortening the length of the protected RNA. In contrast, degradation by fl-*Cg*-Dss1 did not depend on the presence of ATP (Supplementary Fig. 9b, c) and a protection pattern of 13-14 nt fragments was observed regardless of the presence of ATP (Supplementary Fig. 9a). The protected RNA was 5-6 nt longer compared with the 5’-fluorescein-labeled ssRNA substrate in the presence of the antibody, indicating that *Cg*-Dss1 alone forms additional interactions with the substrate around the entry site to the catalytic channel. This would be consistent with the presence of a positively charged patch on the HTH domain (Fig. 1b, c) which could bind the backbone of the RNA.

Supplementary References

1. Pei, J.M., Kim, B.H. & Grishin, N.V. PROMALS3D: a tool for multiple protein sequence and structure alignments. *Nucleic Acids Research* **36**, 2295-2300 (2008).
2. Pei, J.M., Tang, M. & Grishin, N.V. PROMALS3D web server for accurate multiple protein sequence and structure alignments. *Nucleic Acids Research* **36**, W30-W34 (2008).
3. Rambo, R.P. & Tainer, J.A. Accurate assessment of mass, models and resolution by small-angle scattering. *Nature* **496**, 477 (2013).
4. Gruber, A.R., Lorenz, R., Bernhart, S.H., Neuboock, R. & Hofacker, I.L. The Vienna RNA Websuite. *Nucleic Acids Research* **36**, W70-W74 (2008).
5. Frazao, C. et al. Unravelling the dynamics of RNA degradation by ribonuclease II and its RNA-bound complex. *Nature* **443**, 110-4 (2006).
6. Dmochowska, A., Golik, P. & Stepien, P.P. The novel nuclear gene DSS-1 of Saccharomyces cerevisiae is necessary for mitochondrial biogenesis. *Curr Genet* **28**, 108-12 (1995).
